# Targeting scapular muscles with facilitatory movement patterns to improve upper extremity function in gangliocapsular stroke: a case report

**DOI:** 10.1186/s13256-024-04929-x

**Published:** 2024-12-07

**Authors:** Akshaya Saklecha, Moh’d Irshad Quershi, Raghumahanti Raghuveer, Pallavi Harjpal

**Affiliations:** Department of Neurophysiotherapy, Ravi Nair Physiotherapy College, Datta Meghe Institute of Higher Education and Research, Sawangi (Meghe), Wardha, Maharashtra 442001 India

**Keywords:** Scapular alignment, Upper extremity functions, Hemiplegia, Proprioceptive neuromuscular facilitation, Stroke

## Abstract

**Background:**

Gangliocapsular stroke is a specific type of hemorrhagic stroke that primarily affects the basal ganglia and internal capsule due to rupture of the lenticulostriate perforating arteries. Patients afflicted with this condition often present with contralateral motor deficits, particularly affecting the upper extremity. Additionally, these individuals may experience challenges in coordination and stability, largely due to the involvement of the shoulder and scapular girdle. The stability of proximal joint is essential to increase the limited functions and distal movement of the upper extremities. Malalignment of scapula further complicates the execution of daily activities, profoundly affecting patients’ overall well-being and reducing engagement in society. Therefore, restoring upper limb function is imperative for a successful return to daily routines, with a focus on improving arm function being a crucial aspect of stroke therapy. Many methods have been explored for enhancing the function of upper limb in stroke, but the emphasis is more toward developing independence in grasp and improving scapular stability is often neglected. This case reports aim to examine the effect of targeted scapular proprioceptive neuromuscular facilitation on upper limb function in a patient who suffered a gangliocapsular stroke.

**Case presentation:**

A 54-year-old South Asian man was presented with left-sided hemiplegia following a right gangliocapsular stroke. He reported challenges in using his left upper limb and weakness of the left side of the body, which severely impaired his ability to perform activities of daily living. Radiological findings indicated a right gangliocapsular hemorrhage. The patient underwent a 4-week physiotherapy rehabilitation program, with outcome measures including the palpation meter, Fugl–Meyer assessment of upper extremity, and functional independence measure. Following the treatment period, significant improvements were observed in scapular alignment and upper limb functions, underscoring the effectiveness of rehabilitation strategies for optimal outcomes and recovery.

**Conclusions:**

The study underscores the beneficial outcomes of targeted scapular muscles through facilitatory movement patterns to improve upper extremity function in gangliocapsular stroke. Implementing scapular proprioceptive neuromuscular facilitation techniques led to a beneficial change in scapular positioning, consequently improving upper limb function, and quality of life significantly.

## Introduction

Hemorrhagic stroke, accounting for 10–20% of all strokes annually, occurs when a blood vessel in the brain ruptures, leading to bleeding within the brain tissue [1]. A gangliocapsular hemorrhagic stroke is a catastrophic neurological event characterized by the rupture of a blood vessel within the gangliocapsular region of the brain, encompassing the basal ganglia and internal capsule. These structures play a pivotal role in regulating motor control, sensation, and a host of other vital neurological functions. The rupture of a vessels in this area results in the extravasation of blood into the brain parenchyma, precipitating direct tissue damage and secondary effects due to increased intracranial pressure [2].

Chronic hypertension is the primary etiological factor in the majority of these cases, as prolonged elevated blood pressure leads to the degeneration and weakening of small, deep-penetrating arteries, particularly the lenticulostriate arteries originating from the middle cerebral artery (MCA). These vessels are highly susceptible to rupture under sustained hypertensive stress. Additional causes include cerebral amyloid angiopathy, which involves the deposition of amyloid proteins in the walls of cerebral vessels, rendering them brittle and prone to hemorrhage, as well as arteriovenous malformations (AVMs) and aneurysms, both of which represent abnormal vascular formations that are vulnerable to rupture [3].

Clinically, gangliocapsular hemorrhagic stroke manifests with an abrupt onset of severe neurological symptoms. Patients often present with an excruciating headache, frequently described as the most intense they have ever experienced [4].

This is rapidly followed by profound motor deficits, such as hemiparesis or hemiplegia, where one side of the body becomes weak or paralyzed, corresponding to the contralateral side of the brain where the hemorrhage has occurred. Sensory deficits, including loss or alteration of sensation on the affected side, are also common. The hemorrhage exerts significant pressure on surrounding brain structures, leading to alterations in consciousness that can range from confusion to deep coma. Speech disturbances, such as dysarthria or aphasia, are often observed, particularly when the dominant hemisphere is involved. Additional symptoms may include nausea, vomiting, and seizures, driven by the increasing intracranial pressure and the irritation of brain tissue by the extravasated blood [5].

Hemiplegia, a form of paralysis that impacts only one side of the body, is commonly categorized as right or left hemiplegia based on the affected side. According to the National Stroke Association, “nine out of ten patients suffer some degree of paralysis immediately after a stroke.” This may be due to various reasons like pain, subluxation, tightness, and muscle weakness leading to altered position of scapula. This can lead to a reduction in upper limb function. These dysfunctions contribute to difficulties in performing activities of daily living (ADLs) [6].

There are several physiotherapy strategies that can be used to reduce spasticity and enhance upper limb function and scapular alignment. Rood’s Approach, Bobath Neuro Development Treatment, Brunnstrom Movement Therapy, and proprioceptive neuromuscular facilitation (PNF) techniques are a few examples of neurophysiological strategies. All approaches work according to its own principles for restoring voluntary control and mobility [7]. Conventional physiotherapy includes stretching, weight-bearing, and active movement sequences to promote functionality. Activation of the proximal shoulder girdle including scapular muscles will be beneficial in improving the function of the hemiplegic upper limb.

PNF is a neurological method that uses diagonal movement patterns aimed at improving neuromuscular control and performance through techniques that enhance motor control. This approach involves techniques like facilitation, inhibition, strengthening, and relaxation of muscles to reinforce functional movements [8]. Key features of PNF include using diagonal movement patterns and applying sensory cues such as proprioceptive, visual, auditory, and tactile stimuli to enhance or provoke motor responses [9]. The current case report seeks to examine the effects of a scapular PNF on upper limb function in a patient suffering from hemorrhagic stroke.

## Case presentation

A 54-year-old South Asian man, presented at a tertiary healthcare facility with a history of hemorrhagic stroke, following which he had a left-sided hemiparesis. Three moths prior to this presentation, the patient had been in his usual state of health when he suddenly lost consciousness while driving, leading to a fall from his motorcycle. He was subsequently admitted to a tertiary healthcare center in Nagpur, where comprehensive investigations confirmed the diagnosis of a hemorrhagic stroke. The condition was managed medically, yet the patient continued to experience significant difficulties, particularly in utilizing the left side of his body.

Due to persistent challenges, including increased difficulty in performing daily activities and impaired mobility, the patient sought further evaluation and treatment at another tertiary healthcare center in Sawangi, Wardha. He had a known history of hypertension for three years, with a record of inconsistent adherence to prescribed medications. The stroke predominantly affected the upper limbs, severely compromising his ability to perform activities of daily living (ADLs) and ambulate independently.

The patient’s family history revealed no significant genetic predisposition to stroke or other neurological disorders. Psychosocially, he struggled to adapt to the functional limitations imposed by the stroke, which had a profound impact on his quality of life.

### Course

A detailed timeline of events related to the patient's condition are outlined in Table 1.

**Table 1:** Timeline of events

Date	Events
18 May 2023	Sudden fall from the bike due to loss of consciousness around 7:30 pm
18 May 2023	Admitted to a tertiary healthcare center in Nagpur
19 May 2023	Investigations were done and diagnosed as a hemorrhagic stroke
26 May 2023	Got discharged from tertiary healthcare center
11 august 2023	Visited to a tertiary healthcare center in Sawangi Meghe, Wardha
11 august 2023	Referred to neurophysiotherapy

## Clinical findings

The patient provided written consent before the assessment. The patient was examined in supine lying position, and was conscious and well-oriented to time, place, and person. On observation, the left shoulder was depressed, and in adduction, internal rotation and the elbow was flexed and the forearm was pronated. During physical examination, the vital signs were normal. On neurological examination, the mini-mental scale examination score was 28/30. All cranial nerves were intact. Sensory examination revealed that the superficial, deep, and cortical sensations were intact bilaterally over the upper and lower limbs, as well as the trunk. On motor examination, the left upper limb tone was increased as shown in Table 2. Deep tendon reflexes are described in Table 3. At the time of rehabilitation, palpation meter (PALM) is used to assess scapular alignment. Fugl–Meyer assessment of upper extremity (FMA–UE) score was 24/66, indicating severe. The Functional Independence Measure (FIM) score was 99 points.

**Table 2:** Muscle tone according to Modified Ashworth Scale (MAS)

		Right	Left
Shoulder	Flexors	0	1 +
	Extensors	0	1
	Abductors	0	1 +
	Adductors	0	1
Elbow	Flexors	0	1
	Extensors	0	1
Wrist	Flexors	0	1
	Extensors	0	0
Hip	Flexors	0	0
	Extensors	0	0
Knee	Flexors	0	0
	Extensors	0	0
Ankle	Dorsiflexors	0	0
	Plantarflexors	0	0

**Table 3:** Reflexes

	Biceps jerk	Triceps jerk	Supinator jerk	Knee jerk	Ankle jerk	Plantar response
Right	+ +	+ +	+ +	+ +	+ +	Flexor
Left	+ + +	+ + +	+ + +	+ +	+ +	Flexor

### Diagnostics assessment

**Fig. 1:**
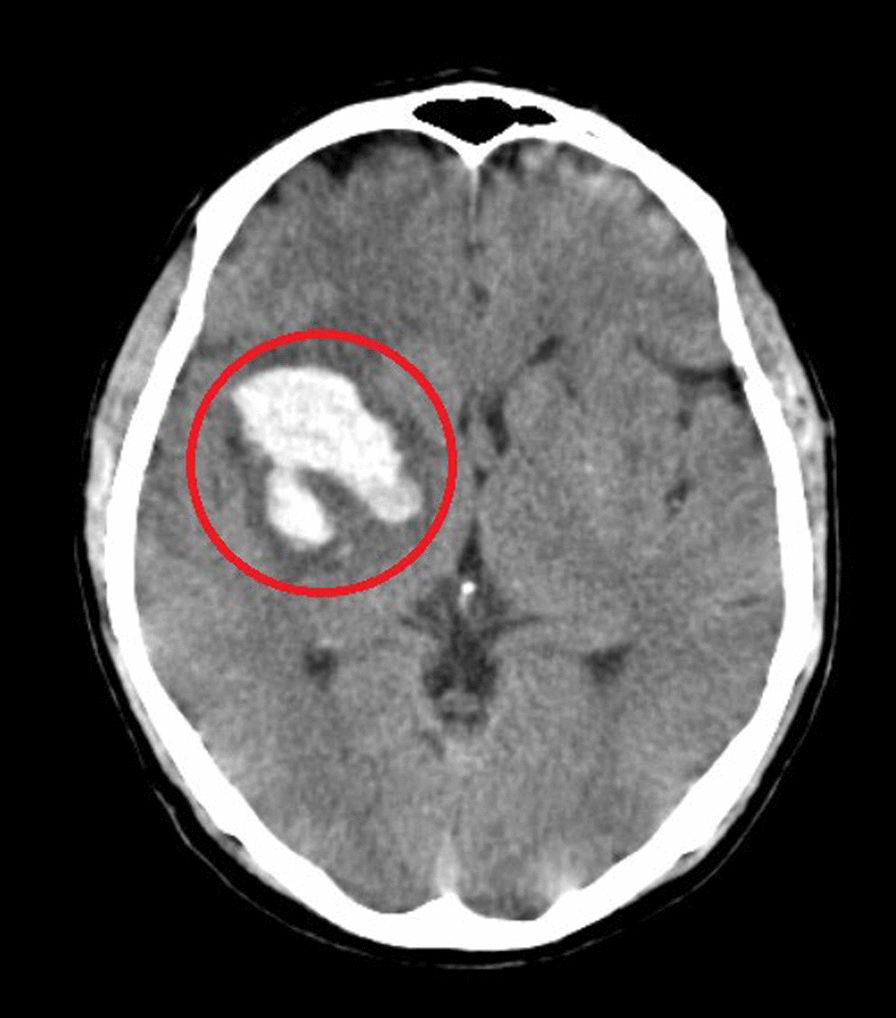
CT brain showing a moderate size of bleed in right gangliocapsular region with involvement of the internal capsule, obligating to the anterior pole of the right lateral ventricle

The diagnostic assessment primarily involved computed tomography (CT) imaging to evaluate the patient's condition post-hemorrhagic stroke. The computed tomography (CT) scan results indicated a right gangliocapsular bleed with involvement of the internal capsule, obligating to the anterior pole of the right lateral ventricle (Fig. 1). The patient faced difficulties accessing timely diagnostic testing post-hemorrhagic stroke, potentially delaying treatment. This finding suggested a gangliocapsular hemorrhage, leading to the diagnosis of left hemiplegia secondary to hemorrhagic stroke.

## Therapeutic intervention

The physiotherapy intervention was implemented over a 4-week period, with sessions scheduled 5 days a week. The treatment plan will integrate scapular and upper limb PNF techniques alongside conventional physiotherapy methods.

For the PNF techniques, diagonal patterns of D1 and D2 flexion and extension were employed targeting scapular and upper limb musculature. These techniques were administered with the patient positioned in side-lying then progressed to sitting posture. The PNF protocol will commence with rhythmic initiation and progress to a repeated contraction [10]. In the context of scapular PNF, specific muscles will be engaged in each diagonal pattern discussed in Table 4. Additionally, conventional physiotherapy will be implemented concurrently discussed in Table 5. The physiotherapy regimen was administered for 60 min per day, 5 days a week, over the 4-week period.

**Table 4:** Scapular proprioceptive neuromuscular facilitation technique

Diagonal patterns	Technique	Dosage
1. Anterior elevation (muscles: levator scapulae, rhomboids, serratus anterior)	Rhythmic initiation and repeated contractions are facilitation techniques applied across all movement patterns 1) Rhythmic initiation: This technique involves teaching the motion in a rhythmic manner, helping the patient to relax, improving coordination, and normalizing the movement pattern 2) Repeated contractions: This technique involves performing repeated contractions to increase active range of motion and strength, guiding the patient’s motion toward the desired pattern of movement	Two sets of ten repetitions 5 days/week for 4 weeks Ample rest periods were incorporated between sets to prevent fatigue and optimize recovery
2. Posterior depression [muscles: latissimus dorsi, serratus anterior (lower), rhomboids]

**Table 5:** Conventional physiotherapy treatment protocol

Exercises category	Exercises	Muscles involved	Dosage
Closed kinematic chain exercises for the upper limb	Wall push-ups Seated push-ups Step-ups using a low platform	Deltoids, pectoralis, triceps, biceps, latissimus dorsi	3 sets of 10–15 repetitions (reps), 3–4 times per week
Range of motion exercises for both upper and lower extremities	Passive and active assisted exercises	Shoulder muscles, elbow flexors and extensors, hip flexors and extensors	10–15 reps per joint, 3–4 times per week
Stretching and strengthening exercises focusing on upper limb, trunk, and lower limb muscles	Hamstring stretches, calf stretches, shoulder stretches Leg press, biceps curls, triceps extensions	Quadriceps, hamstrings, gluteal, abdominals, erector spinae, biceps	Stretch: hold for 30 seconds, three times per muscle groups Strengthen: 3 sets of 10–15 reps, 3–4 times per week
Balance and coordination exercises	Standing with narrow base of support, tandem walking, use of a balance board	Core muscles, lower limb stabilizers (ankle and hip muscles)	15–20 min, three to four times per week
Manual dexterity exercises such as grasp-release activities	Grasp-release activities with small objects, finger opposition exercises, pegboard tasks	Intrinsic hand muscles, flexor and extensor muscles of the forearm	10–15 min per session, three to four times per week
Teaching of activities of daily living (ADLs) tailored to the patient’s individual needs [11]	Dressing, grooming, feeding	Various muscle groups depending on the specific ADLs task	Daily practice incorporated into routine activities

## Follow-up and outcome of interventions

The follow-up was taken on the 4th week of the rehabilitation and there was a commendable improvement in the patient post-rehabilitation. In Table 6, findings of muscle tone of the affected side were shown and in Table 7, pre- and post-rehabilitation scores of palpation meter, FMA–UE and Functional Independence Measure score.

**Table 6:** Muscle tone (graded by the Modified Ashworth Scale)

Pre-rehabilitation left		Post-rehabilitation left
1 +	Shoulder flexors	0
1	Shoulder extensors	0
1 +	Shoulder abductors	0
0	Shoulder adductors	0
1	Elbow flexors	0
0	Elbow extensors	0
1	Wrist flexors	0
0	Wrist extensors	0
0	Hip flexors	0
0	Hip extensors	0
0	Knee flexors	0
0	Knee extensors	0
0	Ankle dorsiflexors	0
0	Ankle plantar flexors	0

**Table 7:** Outcome measures

Outcome measures	Pre-rehabilitation	Post-rehabilitation
1. Palpation meter		
Height discrepancy (in cm)	1.3 cm	0.7 cm
Height discrepancy (in inches)	0.5 inches	0.28 inches
2. FMA–UE	24/66	58/66
3. Functional Independence Measure	99/126	119/126

## Discussion

This case report examined the effects of scapular PNF on scapular position and upper extremity function in a patient with subacute hemiplegia. Hemorrhagic stroke is the subtype of stroke caused by bleeding into the brain as a result of a blood vessel rupture. It is one of the primary causes of impairments in adults with hypertension, often resulting in the loss of motor function. This can lead to a reduction in upper limb function and disturbances in scapular alignment.

Scapular misalignment can cause reduced functional activity in the upper extremities. In turn, upper extremity paresis in patients who suffered from a stroke frequently impairs everyday activities and social roles. Scapular misalignment can be caused by weakening in the serratus anterior and rhomboid muscles. The serratus anterior contributes significantly to scapular stability by producing upward rotation and protraction of the scapula. Rhomboids stabilize the scapula by causing it to retract and adduct [12]. Therefore, in this study, a scapular PNF exercise was conducted for scapular alignment and upper limb functions in subacute hemiplegia.

After 4 weeks of intervention, there was improvement in tone, distance between scapular medial border and spinous process of T3 decreased measured by PALM meter, upper limb function measured by FMA–UE and quality FIM score.

Poonam Chaturvedi *et al*. investigated the mechanism of PNF in stroke. The different PNF techniques facilitate impulses to the brain, and the repetition of patterns has a long-term effect, aiding in the further facilitation of maximum impulses. The aggregate of these impulses generates a motor response and enhances tone in weaker muscles [13].

Another study on hemiplegics investigated the effect of the scapular hold–relax technique on shoulder pain. The control group got only normal treatment, which consisted of 12 sessions of passive shoulder range of motion exercises, stretching exercises, and transcutaneous electrical nerve stimulation (TENS) on 30 stroke survivors with hemiplegia (4 sessions per week). The reduction in shoulder discomfort reported in both groups demonstrates the effectiveness of traditional treatments (such as passive range of motion exercises, stretching exercises, and TENS) in treating hemiplegic patients. Clinical tests demonstrated that the experimental group saw more shoulder improvement. As a result, it claims that scapular PNF has a beneficial effect [14].

Joshi *et al*. [15] studied the effect of scapular PNF on shoulder discomfort, range of motion (ROM), and upper extremity (UE) function in hemiplegic patients, which included thirty individuals. The study found that strengthening of the proximal UE muscles, which helps to correct scapular alignment and improve UE function in patients wgo suffered from a stroke, had a positive influence on post-stroke shoulder discomfort and range of motion [15].

In this study, scapular PNF was utilized in two diagonal patterns: anterior elevation with posterior depression, and posterior elevation with anterior depression. Both patterns were accompanied by two facilitation techniques they are rhythmic initiation and repeated contractions. By integrating scapular PNF techniques into the rehabilitation program, improvements in scapular alignment and upper limb functions were observed after a 4-week intervention. The findings underscore the importance of addressing scapular stability alongside traditional stroke therapy to enhance overall upper limb function, emphasizing the potential of PNF techniques to positively impact scapular positioning and subsequent upper extremity function in stroke rehabilitation. Future studies must be conducted with larger sample size targeting the outcomes for scapular mobility and stability of scapula in gangliocapsular stroke.

## Conclusion

This case report intends to highlight the role of targeting scapular muscles with facilitatory movement patterns to improve upper extremity function in subacute hemiplegia. Implementing scapular PNF techniques led to a beneficial change in scapular positioning, consequently improving which in upper limb function and quality of life significantly.

## Data Availability

All data underlying the results are available as part of the article and no additional source data are required.

## Abbreviations

PNF: Proprioceptive neuromuscular facilitation
PLAM: Palpation meter
FMA-UE: Fugl-meyer assessment of upper extremity
FIM: Functional Independence Measure score
UE: Upper extremity
ROM: Range of motion
TENS: Transcutaneous electrical nerve stimulation
ADLs: Activities of daily living
